# Optimizing allocation for a delayed influenza vaccination campaign

**DOI:** 10.1371/currents.RRN1134

**Published:** 2009-12-13

**Authors:** Jan Medlock, Lauren Ancel Meyers, Alison Galvani

**Affiliations:** ^*^Department of Mathematical Sciences, Clemson University and ^†^The University of Texas at Austin

## Abstract

During unexpected infectious disease outbreaks, public health agencies must make effective use of limited resources. Vaccine distribution may be delayed and staggered through time, as underscored by the 2009 H1N1 influenza pandemic. Using a mathematical model parametrized with data from the 2009 H1N1 pandemic, we found that optimal allocations of vaccine among people in different age groups and people with high-risk conditions depends on the schedule of vaccine availability relative to the progress of the epidemic. For the projected schedule of H1N1 vaccine availability, the optimal strategy to reduce influenza-related deaths is to initial target high-risk people, followed by school-aged children (5–17) and then young adults (18–44). The optimal strategy to minimize hospitalizations, however, is to target ages 5–44 throughout the vaccination campaign, with only a tiny amount of vaccine used on high-risk people. We find that optimizing at each vaccine release time independently does not give the overall optimal strategy. In this manuscript, we derive policy recommendations for 2009 H1N1 vaccine allocation using a mathematical model. In addition, our optimization procedures, which consider staggered releases over the entire epidemic altogether, are applicable to other outbreaks where not all supplies are available initially.

## Introduction

 Vaccines are critical to controlling the spread of influenza. The development and distribution of effective vaccines for new pandemic strains may take several months, and not all the vaccine doses may be available at the same time. In addition, prior to and throughout the vaccination period, a substantial fraction of the population may become infected and thereby naturally immunized to re-infection. These dynamics change the transmission pattern and need to be considered in order to optimize vaccine allocation.

 Such changing dynamics are also the case for the 2009 H1N1 influenza pandemic. The first 2009 H1N1 vaccines were distributed in the US in early October mainly to health care personnel and young children [1]. The Centers for Disease Control and Prevention (CDC) projected that a total of 45 million doses would be available by mid-October, followed by 20 million doses every week thereafter [2]. The optimal allocation of influenza vaccines when vaccination is delayed and staggered throughout the epidemic period is critical to public health, yet may differ substantially from the optimal allocation of vaccines prior to an epidemic. Here we evaluate age-specific and risk-specific vaccine allocation strategies according to the CDC’s projected 2009–2010 schedule of vaccine availability for 2009 H1N1.

 Public health agencies often consider projected impacts on deaths and hospitalizations when determining intervention policy [3]. Severe infections that require hospitalization have the potential to exceed the capacity of health services, particularly at the peak of an outbreak. Averting deaths is also clearly a prime humanitarian goal. We determine the optimal allocation of vaccines according to both of these measures by employing an optimization procedure that explicitly incorporates the transmission dynamics of 2009 H1N1 influenza prior to and throughout the vaccination period.

 Our analysis indicates that both deaths and hospitalizations can be minimized by prioritizing the vaccination of school-aged children (5–17), young adults (18–44), and people at high-risk of severe infections. However, the temporal order of prioritization differs between these two outcome measures when staggered vaccination schedules are considered. Deaths can be minimized by prioritizing high-risk people first, followed by schoolchildren and young adults. In contrast, hospitalizations are optimally averted by first prioritizing schoolchildren and young adults, followed by high-risk people. In contrast to both optimal strategies, the CDC recommends prioritizing high-risk people and people aged 6 months through 24 years [4]. Both deaths and hospitalizations for the 2009 H1N1 pandemic could be reduced by shifting focus to schoolchildren and young adults. 

## Results

 To minimize deaths, the optimal policy is for the first two batches of vaccine to be allocated to high-risk people, followed by schoolchildren (ages 5–17) and then young adults (ages 18–44) (Figure 1A). To minimize hospitalizations, the first five batches should be allocated to schoolchildren and young adults, with a small amount of vaccine also allocated to high-risk people in the fifth batch (Figure 1B).

**Figure:**
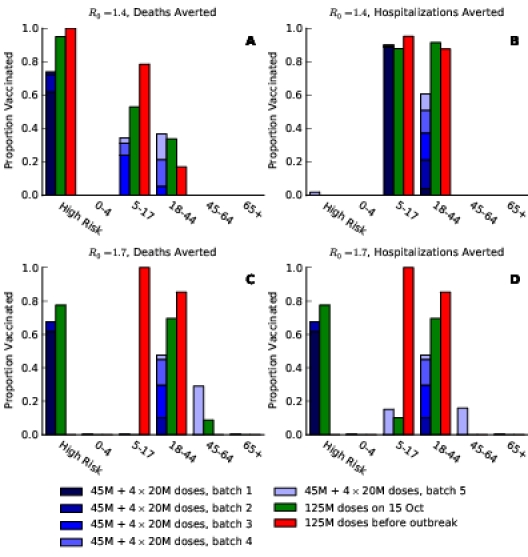
Figure 1. Optimal vaccine allocation for different vaccine delivery schedules, different values of \begin{equation*}R_0\end{equation*}, and different objectives.  The delivery schedule "45M + 4 × 20M doses" corresponds to five batches of vaccine delivery: 45M doses on 15 October and 20M doses each of the following four weeks.  The different shades of blue correspond to the optimal allocation of the different delivery batches. The green and red bars indicate the optimal allocations under hypothetical early delivery scenarios of 125M doses on 15 October and prior to the outbreak, respectively.

As the basic reproductive number (\begin{equation*}R_0\end{equation*}) increases, optimal allocation to minimize deaths no longer includes schoolchildren (Figure 1C), while optimal allocation for hospitalizations shifts to include high-risk people (Figure 1D). This is due in part to acquired immunity: for \begin{equation*}R_0 = 1.4\end{equation*}, only 11% of schoolchildren have been infected before the first vaccine is available on 15 October, whereas, for \begin{equation*}R_0 = 1.7\end{equation*}, 45% of schoolchildren have been infected by 15 October.

 The optimal allocation is sensitive to the scheduling of release times. Consequently, evaluation of each batch separately does not generate the allocation strategy that is optimal overall (see Text S1 and Figures S3–S10). For example, when only two total batches are available (45 million doses on 15 October and 20 million on 22 October), deaths can be minimized by allocating the first batch to schoolchildren and the second batch to adults aged 18–44 (Figure S3). In contrast, when 3 total batches are available (45 million doses on 15 October and 20 million on each of 22 October and 29 October), the first two batches are optimally split between schoolchildren and high-risk people and the third batch is used exclusively on high-risk people.

 The projected reductions in infections, deaths, and hospitalizations are substantial for both sets of optimized vaccination strategies (Figure 2). The strategy that minimizes hospitalizations is predicted to reduce the total attack rate more effectively than the strategy that minimizes deaths (11.4% versus 15.2% of the population infected, respectively). Comparing the number of deaths and hospitalizations, the strategy that minimizes deaths results in 8000 fewer deaths (a 23% reduction) and 44,000 more hospitalizations (a 9% increase) than the strategy that minimizes hospitalizations. Since the strategy that minimizes deaths also greatly reduces hospitalizations, it may be preferred by decision makers.

**Figure:**
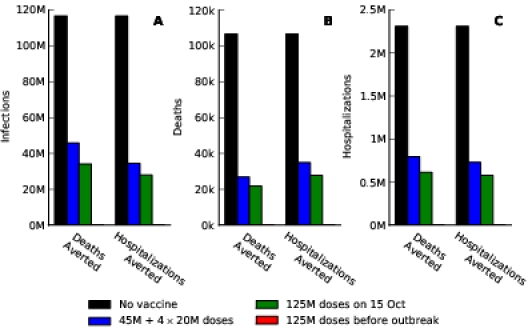
Figure 2. The impact on infections, deaths, and hospitalizations for different vaccine delivery schedules and different objectives, with \begin{equation*}R_0 = 1.4\end{equation*}.

 The staggered vaccine delivery schedule that we considered had a total of 125 million doses in five batches starting on 15 October. To explore the effect of the delay in vaccine delivery, we also calculated the optimal allocations when all 125 million doses were delivered in one batch, either on 15 October or before the outbreak began (Figure 1). The epidemiological impact of the initial vaccination delay is substantial: the staggered release results in 23% more deaths and 26% more hospitalizations, respectively, compared to optimal allocation of the complete distribution in October. By contrast, the optimal distribution of 125 million doses before the outbreak begins prevented the outbreak entirely (Figure 2).

## Discussion

 Vaccination not only protects those who are vaccinated, but also reduces transmission to others in the population. Schoolchildren, and secondarily young adults, are responsible for most of the influenza transmission [5] [6] [7] [8] [9] [10], although this may shift late in a pandemic after a large fraction of children have already become immune [11]. It has been previously demonstrated that the vaccination of these age classes can optimize multiple outcome measures, including infections averted, deaths averted, economic cost, and years of life saved [12]. However, it was previously assumed that all available vaccines were allocated prior to the outbreak. As is the case for the current 2009 H1N1 pandemic, vaccines may not become available until the midst of an outbreak requiring critical policy decisions under dynamic conditions. Indeed, we find that the temporal dynamics of influenza transmission and vaccine availability affect the optimization of vaccine allocation, and that the optimal allocation changes through time. For example, the importance of reducing transmission declines throughout the outbreak and thus so does the value of vaccinating individuals based on their potential for transmission.

 We evaluated the optimal age-specific and risk-specific allocation of 2009 H1N1 influenza vaccine doses based on the schedule of vaccine availability projected by the CDC. In general, our results suggest that low-risk children between 6 months and 4 years of age and adults 65 and older should receive the lowest priority to minimize both hospitalizations and deaths. These groups are the least responsible for transmission, and are estimated to have relatively low case mortality ratios for 2009 H1N1 [13]. Although hospitalization ratios are higher in high-risk people, vaccination of low-risk schoolchildren and young-adult age classes is most effective at minimizing hospitalizations, via reducing transmission, thereby indirectly protecting high-risk people. For staggered vaccine release, after a large fraction of schoolchildren and young adults have been vaccinated, adults aged 45–64 years should then be vaccinated. In order to reduce deaths, high-risk people should be given priority, followed by the schoolchildren and then young adults. Deaths are minimized by protecting the high-risk group directly.

 Despite the current stage of the 2009 H1N1 outbreak, the vaccination of those most responsible for transmission remains important. For diseases that are more transmissible than the current 2009 H1N1 influenza, and particularly if vaccination is not available early on during the outbreak, control of the outbreak via the reduction of transmission becomes very difficult, increasing the importance of vaccinating high-risk people (e.g. Figures S1 & S2 for \begin{equation*}R_0 = 2.0\end{equation*}).

 The CDC issued 2009 H1N1 vaccine guidelines in late July 2009 [4], which prioritized high-risk people (including pregnant women) and people aged 6 months through 24 years, in addition to health-care workers and the contacts of children under 6 months of age. Our analysis suggests a different strategy is optimal. If the public health objective is to minimize deaths due to 2009 H1N1, earliest priority should be given to high-risk people, followed by school-aged children and then young adults (18–44 years old). In contrast, if the public health objective is to minimize hospitalizations, only school-aged children and then young adults, with only a small fraction of the supply finally going to high-risk people. These optimal vaccine allocations differ from the CDC recommendations by excluding children aged 6 months to 4 years and including adults aged 25–44. Even under the early vaccine delivery scenarios, we find that priority should still be given to high-risk people, schoolchildren and young adults, and not to individuals aged 6 months to 4 years and 25–44 years. 

 We have found that the entire epidemic period must be considered in order to determine optimal vaccine allocations when availability is staggered. In contrast to traditional epidemic models, our optimization methodology allows the scheduling of vaccine availability to be taken into account. The methodology developed here is broadly applicable. It can be used for other infectious-disease outbreaks in which not all supplies, whether vaccines or drugs, are available at the start of the epidemic. 

## Methods

 We extended the age-structured SEIR (susceptible, latent, infectious, recovered) model of Medlock & Galvani [12] to include two levels of risk for complications due to influenza infection. We parametrized this model using data from the 2009 H1N1 influenza pandemic. We also extended the framework to incorporate staggered delivery of vaccine doses. We outline the methods here; see Text S1 for more details.

 We assumed that the mean latent period was 2 days [14] [15] and that the mean infectious period was 7 days [14]. We also assumed that people aged 50 and older had some residual immunity due to antigenic similarity of 2009 H1N1 with other H1N1 viruses circulating in the past [4] [16]. The contact matrix describing potential infectious contacts between individuals of different ages was taken from survey data [12] [17], with the probability of infection given a sufficient contact being chosen so that the basic reproductive number (\begin{equation*}R_0\end{equation*}) was \begin{equation*}1.4\end{equation*} [18]. We also considered \begin{equation*}R_0 = 1.2\end{equation*}, \begin{equation*}1.7\end{equation*}, & \begin{equation*}2.0\end{equation*} (Text S1).

 The transmission model was parametrized with a mean of 22.5% of the population defined as at high risk for influenza complications, due to other, preexisting medical conditions, with the proportion at high risk varying among age groups [19]. We assumed that high-risk people had 9 times higher case mortality ratio and 3 times higher case hospitalization ratio, compared to low-risk people [20]. For the age-dependent case mortality ratios and case hospitalization ratios, averaged over risk, we used the ratios derived in a statistical analysis of deaths, hospitalizations, and survey-reported H1N1 infections from New York City and Milwaukee [13].

## Supporting Material 

Text S1: Detailed Methods

**Figure:**
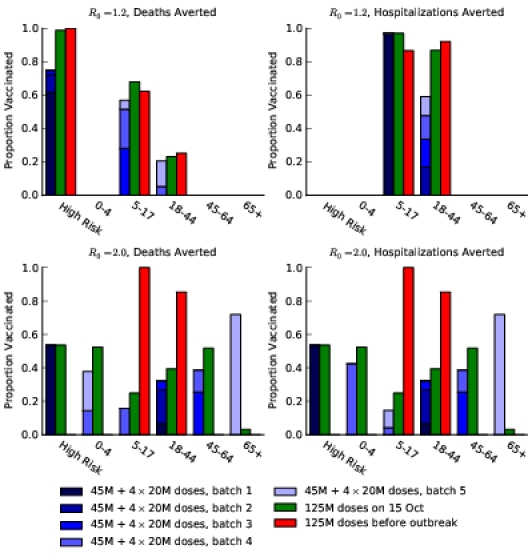
Figure S1. Optimal vaccine allocation for different vaccine delivery schedules, different values of \begin{equation*}R_0\end{equation*}, and different objectives.  The delivery schedule "45M + 4  20M doses'' corresponds to 5 batches of vaccine delivery: 45M doses on 15 October and 20M doses each of the following 4 weeks.  The different shades of blue correspond to the optimal allocation of the different delivery batches.  The graphs for \begin{equation*}R_0 = 1.4\end{equation*}and \begin{equation*}1.7\end{equation*} are in the main text.

**Figure:**
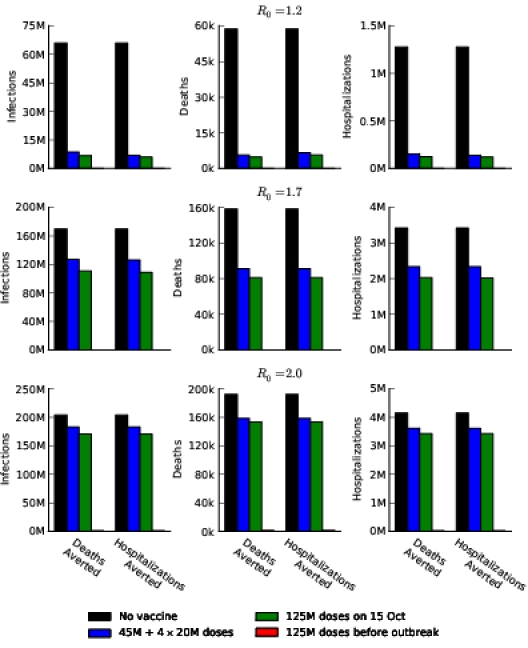
Figure S2. The impact on infections, deaths, and hospitalizations for different vaccine delivery schedules, different values of \begin{equation*}R_0\end{equation*}, and different objectives.  The graphs for \begin{equation*}R_0 = 1.4\end{equation*} are in the main text.

**Figure:**
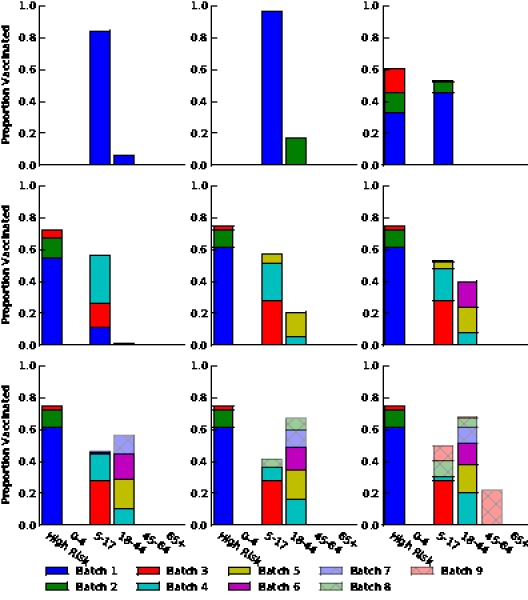
Figure S3. Optimal vaccine allocation for different numbers of vaccine batches for \begin{equation*}R_0 = 1.2\end{equation*} and deaths averted.  The delivery schedules consist of from 1 to 8 batches of vaccine delivered in intervals of 1 week, with the first batch being 45M doses and subsequent batches being 20M doses.  Each graph shows the optimal allocation given the number of batches of vaccine available.

**Figure:**
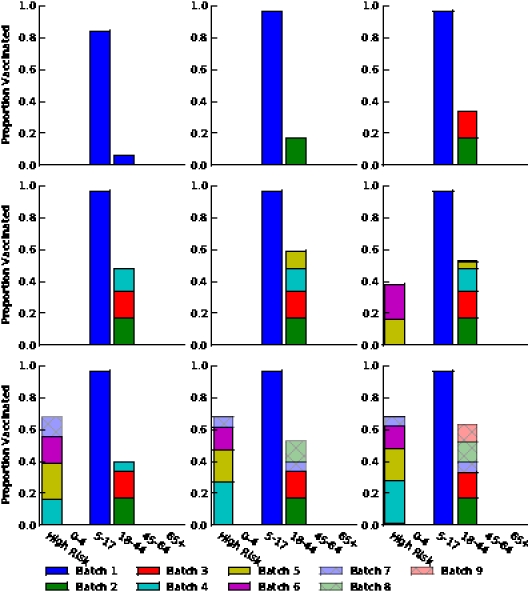
Figure S4. Optimal vaccine allocation for different numbers of vaccine batches for \begin{equation*}R_0 = 1.2\end{equation*} and hospitalizations averted.  The delivery schedules consist of from 1 to 8 batches of vaccine delivered in intervals of 1 week, with the first batch being 45M doses and subsequent batches being 20M doses.  Each graph shows the optimal allocation given the number of batches of vaccine available.

**Figure:**
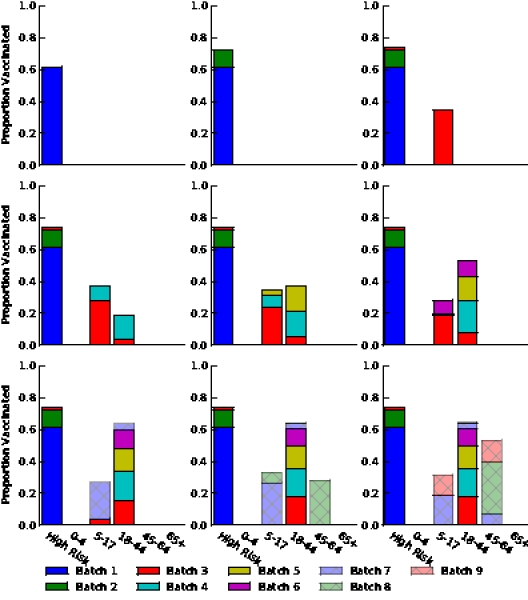
Figure S5. Optimal vaccine allocation for different numbers of vaccine batches for \begin{equation*}R_0 = 1.4\end{equation*} and deaths averted. The delivery schedules consist of from 1 to 8 batches of vaccine delivered in intervals of 1 week, with the first batch being 45M doses and subsequent batches being 20M doses.  Each graph shows the optimal allocation given the number of batches of vaccine available.

**Figure:**
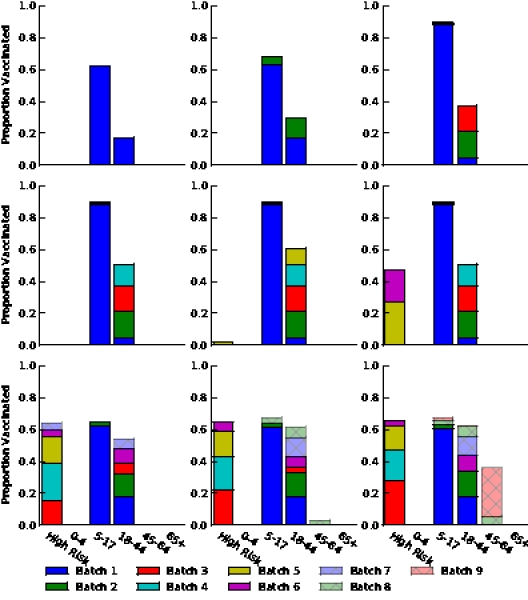
Figure S6. Optimal vaccine allocation for different numbers of vaccine batches for \begin{equation*}R_0 = 1.4\end{equation*} and hospitalizations averted. The delivery schedules consist of from 1 to 8 batches of vaccine delivered in intervals of 1 week, with the first batch being 45M doses and subsequent batches being 20M doses.  Each graph shows the optimal allocation given the number of batches of vaccine available.

**Figure:**
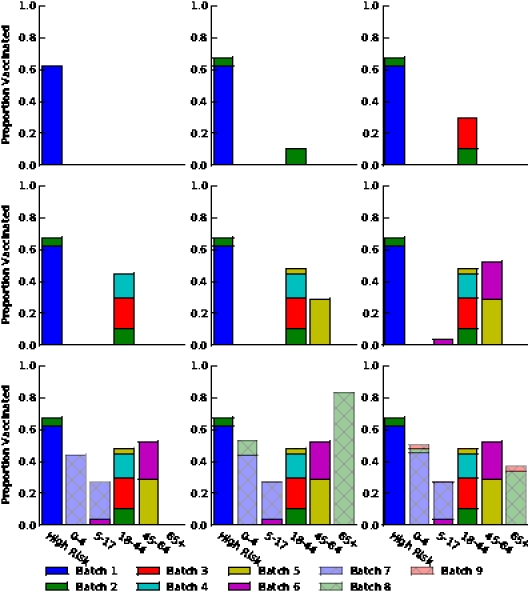
Figure S7. Optimal vaccine allocation for different numbers of vaccine batches for \begin{equation*}R_0 = 1.7\end{equation*} and deaths averted.  The delivery schedules consist of from 1 to 8 batches of vaccine delivered in intervals of 1 week, with the first batch being 45M doses and subsequent batches being 20M doses.  Each graph shows the optimal allocation given the number of batches of vaccine available.

**Figure:**
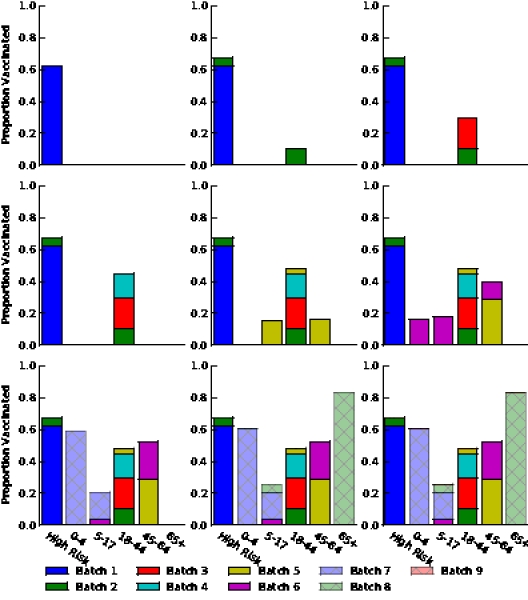
Figure S8. Optimal vaccine allocation for different numbers of vaccine batches for \begin{equation*}R_0 = 1.7\end{equation*} and hospitalizations averted.  The delivery schedules consist of from 1 to 8 batches of vaccine delivered in intervals of 1 week, with the first batch being 45M doses and subsequent batches being 20M doses.  Each graph shows the optimal allocation given the number of batches of vaccine available.

**Figure:**
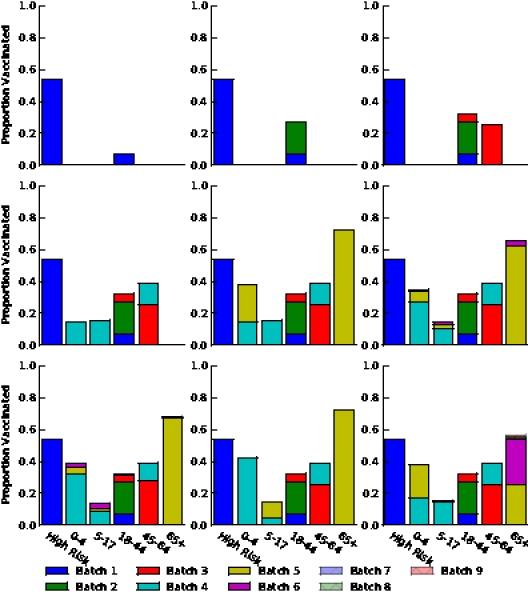
Figure S9. Optimal vaccine allocation for different numbers of vaccine batches for \begin{equation*}R_0 = 2.0\end{equation*} and deaths averted.  The delivery schedules consist of from 1 to 8 batches of vaccine delivered in intervals of 1 week, with the first batch being 45M doses and subsequent batches being 20M doses.  Each graph shows the optimal allocation given the number of batches of vaccine available.

**Figure:**
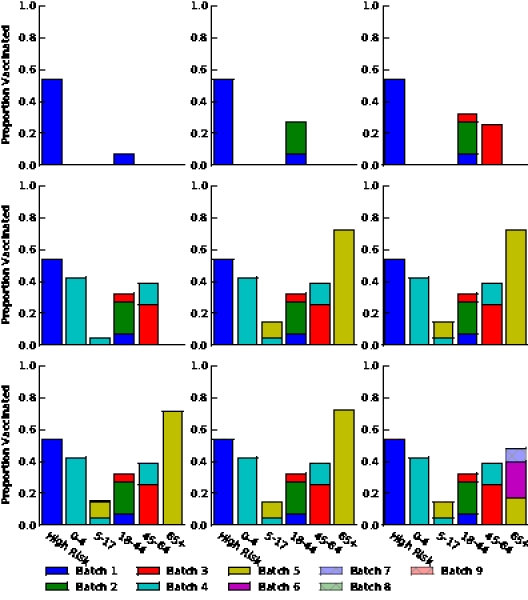
Figure S10. Optimal vaccine allocation for different numbers of vaccine batches for \begin{equation*}R_0 = 2.0\end{equation*} and hospitalizations averted.  The delivery schedules consist of from 1 to 8 batches of vaccine delivered in intervals of 1 week, with the first batch being 45M doses and subsequent batches being 20M doses.  Each graph shows the optimal allocation given the number of batches of vaccine available.

## Funding Information

 Funding was provided in part by NIH grant 1U01GM087719-01.  JM was supported in part by start-up funding from the Clemson University Department of Mathematical Sciences.  The funders had no role in study design, data collection and analysis, decision to publish, or preparation of the manuscript.

## Competing Interests

 All of the authors have no competing financial, personal, or professional interests that have influenced this paper.
